# No evidence for language syntax in songbird vocalizations

**DOI:** 10.3389/fpsyg.2024.1393895

**Published:** 2024-05-02

**Authors:** Gabriël J. L. Beckers, Marinus A. C. Huybregts, Martin B. H. Everaert, Johan J. Bolhuis

**Affiliations:** ^1^Department of Psychology, Cognitive Neurobiology and Helmholtz Institute, Utrecht University, Utrecht, Netherlands; ^2^Institute for Language Sciences, Utrecht University, Utrecht, Netherlands; ^3^Department of Psychology and St. Catharine's College, University of Cambridge, Cambridge, United Kingdom

**Keywords:** birds, combined calls, linear order, Merge, language, evolution

## 1 Introduction

The evolutionary origins of human language remain poorly understood and hotly debated. In a recent study published in *Nature Communications* (Suzuki and Matsumoto, 2022), the authors claim to have found evidence for what they call “Core-Merge” in the vocal communication of Japanese tits (*Parus minor*, a passerine bird species). As the authors suggest that Core-Merge—allowing senders to combine two words and receivers to recognize them as a single unit—is a cognitive capacity underlying human language, their findings would have important implications for the study of the evolution of language (Bolhuis et al., 2014). Here we argue that a role for Core-Merge in language evolution is not evident and that their study does not demonstrate Core-Merge in birds. Instead, we argue that their findings can be explained as differential responsiveness to distinctive vocalizations, based on concatenation of vocal utterances.

Suzuki and Matsumoto (2022) adopt a theoretical approach which takes the concepts of Merge and Compositionality as central to the human language faculty. Merge as first introduced in linguistic theorizing (Chomsky, 1995) is taken as the basic operation for generation of unbounded language. Hierarchical structure—and subsequently deriving meaning based on structure, i.e., compositionality—is at the core of language and is an automatic effect of application of Merge (Berwick and Chomsky, 2016; Chomsky, 2017):


$$\begin{array}{l} (\text{I})\text{ Merge }(\text{x},\text{y})\;=\;\{\text{x},\text{y}\},\\ \text{ where x},\text{ y is either a word-like atom or an object that is itself a product of Merge}. \end{array}$$

To illustrate (I), Merge can take the words *the* and *book* to form the set {*the, book*}, which is now a new element to which Merge may apply. A further application of Merge may then combine that set with *read* to form {*read*, {*the, book*}}. In this way, Merge automatically generates the full range of hierarchical structure that is characteristic of human language and distinguishes it from all other known human and non-human cognitive systems (Everaert et al., 2015). Note that Merge is set formation, generating structure without imposing any order, which is derived by additional mechanisms. This makes sense given the fact that vocalization (spoken languages, birdsong) always introduces order, being temporally bound, but, for instance, sign languages need not “suffer” from this restriction, and allow for other arrangements like visual space.

Crucially, Suzuki and Matsumoto's (2022) claim is based on a line of reasoning in which Merge is stepwise derived with Core-Merge being the first step. We will return to this issue in Section 3.

## 2 Combining calls

In their study, the authors measured the behavioral responses of Japanese tits to different combinations of alert calls (A) and recruitment calls (R). In nature, these birds produce such calls in isolation but also in the combination AR (A followed by R). AR is thought to be used to elicit mobbing behavior, whereas A and R have different functions (Suzuki et al., 2016). Previous playback experiments have indeed shown that the birds respond to A, R, and AR with distinctly different patterns of “scanning” and “approach” behavior, and that they distinguish AR from an artificially reversed RA combination (Suzuki et al., 2016; Bolhuis et al., 2018). In the current study, birds were tested on four stimulus conditions: (1) 1AR, in which an AR vocalization is played from one speaker; (2) 2AR, in which an A vocalization is played from one speaker, and is followed by an R vocalization played from a different speaker 10 m apart; (3) 1RA, an artificial vocalization in which the order of calls is reversed, played from the same speaker; and, (4) 2RA, in which R is followed by A, each from a different speaker. This time a stuffed predator was present and different behaviors were measured, “wing flicks” and “predator approach”. The birds responded with these behaviors only to 1AR, and not to any of the other stimuli, which the authors see as a confirmation of their prediction that “If an animal has evolved core-Merge, then it should be able to distinguish a two-call sequence produced by a single individual from two temporally linked calls produced by multiple individuals”.

We agree that the experiment shows that 1AR can be seen as one utterance (see Schlenker et al., 2023), contrary to 2AR, and that that, in principle, could imply a form of syntax, in particular concatenation. We do not agree, however, with the authors' claim that Core-Merge is the right way to describe this increase in the Japanese tits' repertoire of vocalizations.

## 3 Core-merge

Suzuki and Matsumoto (2022) present Core-Merge as a cognitive capacity that they assume underlies language, although it is in fact not a notion widely established in the literature. They adopt the notion of Core-Merge, and the role it plays in language evolution, following the work of Fujita (2014), summarized in (II):


$$\begin{array}{l} \text{(II) a. Core-Merge:}\;\left(\alpha ,\beta \right)\Rightarrow \left\{\alpha ,\;\beta \right\}\\ \text{ b. Core-Merge}+\text{Recursion}=\text{Recursive Merge} \end{array}$$

The authors thus assume that there are two independent cognitive capacities, Core-Merge and its recursive application, and that together these deliver “Recursive Merge”, what we above have called Merge (I). However, what does it mean to take “recursion” as a separate cognitive capacity? What may its evolutionary trajectory look like? Recursion is a property of a rule system, not a separate cognitive property like executive function or memory. Furthermore, the authors use Core-Merge, which Fujita (2014) presents as set formation (cf. IIa), to account for an animal call system that appears to use, in fact, the linear order of two calls. So, why consider a new operation, called Core-Merge, that has a single application to combine two specific calls without giving linear order, when sensitivity to linear order is what is demonstrated by the results?

Rizzi's alternative notion “1-Merge” (Rizzi, 2016) could be more appropriate for what the authors intend to achieve because, as Schlenker et al. (2016, p. 183–184) observe “1-Merge” is simple concatenation, and such a combinatorial device “should not be taken to involve a real instance of ‘merge'.” The concatenation operation combines two strings *x* and *y* into a longer string *x*^∧^*y*. The elements of the string, and therefore *x, y*, and *x*^∧^*y*, are linearly ordered. Combining A and R into AR is thus plain concatenation of two strings of notes A and R into a novel and longer string of notes AR. In other words, one should simply avoid using the term “Merge” for how “Core-Merge” and “1-Merge” are used in the stepwise evolutionary theory that Fujita suggests, and Suzuki and Matsumoto adopt.

A Merge-based system of derivation, as in (I), involves parallel operations. Thus, if *x* and *y* are merged, each object *x,y* may possibly have been already constructed by a previous Merge operation. So, we must assume there is a workspace, which has access to the lexicon of atomic elements, and contains any new object that is constructed. There is just a single, unified Merge operation, recursive by definition: no half-Merge (Berwick and Chomsky, 2019); no half-recursion (Huybregts, 2019), i.e., no stepwise development of Merge.

## 4 Discussion

We believe core-Merge is an unhelpful notion for comparing the birds' call communication system with human language and suggest a different explanation for the experimental results: 1AR is a natural AR vocalization used in predator mobbing contexts (Suzuki et al., 2016), so the birds respond with mobbing behavior. Based on auditory localization, 2AR is not interpreted as a vocalization but as two vocalizations: one bird calling A and a different bird 10 m apart calling R. Single A and R calls communicate different messages, each of which is different from AR (Suzuki et al., 2016), so the birds do not respond as if it is AR. The same reasoning holds true for 2RA, which is interpreted as an R and an A call, each from a different bird. Lastly, 1RA is interpreted as originating from one sound source, but it is artificially reversed and may not be a natural vocalization, or at least is not known to be used in mobbing contexts, so the birds are not expected to respond to it with mobbing like they do to AR. Taken together, this explanation accounts for the prediction of the authors that AR should be responded to differently from each of the other stimuli.

We thus agree with Suzuki and Matsumoto (2022) that the Japanese tits interpret A, R and AR as three different vocalizations and that to distinguish AR from RA they must somehow be sensitive to the linear order of vocal utterances. Sensitivity to note order within single calls had already been shown in an earlier field study (Suzuki, 2014). The evolution of sensitivity to linear order in such call sequences as AR may well constitute a further step in increasing the possibilities for coding of information in animal communication. This notwithstanding, from the results so far it is not possible to infer *how* the order of notes in these vocalizations are internally represented.

The simplest explanation for the results in Suzuki and Matsumoto (2022) would be a finite listing, in which case the perception of AR would not require any special combinatorial mechanisms (Beckers et al., 2017) but could be based on the same mechanisms that are also required for the perception of the individual A and R calls. Each of these calls are long and complex vocalizations consisting of a sequence of acoustically distinct notes (Suzuki, 2014), and their perception therefore already requires temporal integration of multiple vocal units. To receivers, A, R and AR are then simply three complex, acoustically distinct objects eliciting different behaviors.

In addition, it is difficult to see how the earlier result of Suzuki et al. (2017) is consistent with the latest results (Suzuki and Matsumoto, 2022), in which it is concluded that AR is interpreted as a compound vocalization because it is from one bird. The 2AR sequences with the separate utterances A, R vocalized by two birds are responded to differently. In Suzuki et al. (2017), the birds responded to an artificial AR' call as if it was AR, even though the A and R' calls in AR' must be separate utterances from different birds because they are calls from different species (Schlenker et al., 2023).

Although there thus remain questions as to how to integrate all results into one explanation, we do think it is a strong result that all experiments so far (Suzuki et al., 2016, 2017; Suzuki and Matsumoto, 2022) consistently show sensitivity to the linear order of call utterances, because AR is always different from RA (and AR' from R'A). In view of the earlier results of Suzuki et al. (2017), rather than finite listing, we suggest a concatenation-based, finite state system that can be represented by a set of bigrams (e.g., {#A, #R, AR, A#, R#}). Such an account naturally provides call order.

Taken together, we do not see a convincing case for Merge in human language being based on two separate capacities, Core-Merge and its recursive application, unlike Suzuki and Matsumoto (2022). But irrespective of one's view on this, Fujita's Core-Merge, as adopted by Suzuki and Matsumoto, is set-formation and does not generate linear order, and an explanation of the results in Japanese tits should thus be based on a different operation, in particular concatenation. We do not agree, therefore, with their claim that Core-Merge explains the repertoire increase of vocalizations, or with their suggestion that such call combination could be the first step toward hierarchically structured expressions.

## Author contributions

GB: Writing – original draft, Writing – review & editing. MH: Writing – original draft, Writing – review & editing. ME: Writing – original draft, Writing – review & editing. JB: Writing – original draft, Writing – review & editing.
